# Exploring the Genetic Basis of Ketosis: Preliminary GWAS Findings on Beta-Hydroxybutyrate Levels in Holstein Cattle

**DOI:** 10.1155/ijog/5520648

**Published:** 2025-07-01

**Authors:** Veysel Bay

**Affiliations:** Department of Animal Science, Ege University, İzmir, Türkiye

**Keywords:** dairy cattle ketosis, genome-wide association, QTL

## Abstract

Ketosis is a metabolic condition characterized by a shift in energy metabolism, occurring when glucose availability is depleted and fat becomes the alternative primary energy source, resulting in the accumulation of ketone bodies. In dairy cattle, ketosis represents a significant challenge, adversely affecting both animal health and farm productivity. The genetic basis of ketosis in cattle has attracted increasing attention, with genome-wide association studies (GWAS) emerging as a crucial method for identifying relevant genetic factors. This study was aimed at investigating genome-wide regions associated with beta-hydroxybutyrate (BHB) concentrations in Holstein–Friesian cows' blood before calving in the United Kingdom. BHB measurement data from 253 previously genotyped cows were used in the analyses. The results revealed five significant SNPs on Chromosome 15 (BTA15) and one significant SNP on BTA5 (*p* < 1.60e − 6). Notably, the SNPs on BTA15 clustered within a genomic region enriched with genes implicated in lipid metabolism and energy balance, highlighting its potential role in ketosis susceptibility. These preliminary findings refine the genetic architecture of ketosis and offer new avenues for improving dairy cattle health and welfare through targeted genetic selection programs while highlighting the need for validation in larger and independent populations.

## 1. Introduction

The rising demand for food production has led to intense selection pressures on production animals, increasing the incidence of diseases [1, 2]. Dairy cattle ketosis is one such disease, a metabolic disorder that presents a significant challenge to the dairy industry by affecting animal health and farm productivity [3, 4]. Ketosis predominantly occurs in high-yielding dairy cows during the early lactation phase when energy demands exceed food consumption. This negative energy balance leads to the mobilization of body fat reserves, producing ketones such as beta-hydroxybutyrate (BHB) and acetoacetate [5, 6]. Monitoring ketosis in dairy cattle is crucial for managing herd health and productivity. Ketosis is assessed by evaluating ketone bodies in the blood, milk, or urine. Blood tests measure BHB levels, considered the most accurate indicator of ketosis [7, 8]. Urine tests often use dipsticks that detect acetoacetate, another ketone body, providing a quick and cost-effective screening tool, though they are less precise than blood tests [9]. Milk tests can also detect BHB, and innovations like Fourier-transform infrared spectroscopy (FTIR) offer noninvasive, real-time monitoring of ketone levels [10]. The clinical manifestations of ketosis include reduced milk yield, weight loss, decreased appetite, and, in severe cases, neurological symptoms, collectively leading to substantial economic losses [11, 12].

Recent advances in genetics and genomics offer promising avenues for understanding and mitigating the incidence of ketosis [3, 13]. Genetic predisposition plays a crucial role in an individual cow's susceptibility to ketosis, with heritability estimates for ketosis traits ranging between 0.012 and 0.28 [14, 15]. The low to moderate heritability suggests that selective breeding based solely on phenotypic selection may have limited effectiveness for reducing ketosis susceptibility. However, genomic studies have identified several quantitative trait loci (QTLs) and candidate genes associated with metabolic traits influencing ketosis, which could substantially enhance the precision and efficiency of genetic selection programs [2, 16–18].

Integrating genomic data with traditional breeding programs facilitates the identification of superior genotypes that are less prone to metabolic imbalances [19]. Genomic selection, which utilizes dense marker panels to predict breeding values, enhances the accuracy of selecting animals with favorable genetic profiles for ketosis resistance [15, 20]. Furthermore, the application of functional genomics, including transcriptomics and metabolomics, provides deeper insights into the biological pathways involved in the onset of ketosis [21]. By elucidating the complex interactions between genetic factors and metabolic processes, these approaches pave the way for the development of more targeted and effective interventions [22, 23].

Several genome-wide association studies (GWAS) have been conducted to explore the genetic basis of ketosis in dairy cattle in the United States [2], Canada [17], China [18], and Germany [16], providing valuable insights into the loci and genes involved in this metabolic disorder. The objective of this study was to identify genomic regions associated with BHB concentrations in the blood of Holstein–Friesian cows prior to calving in the United Kingdom.

## 2. Materials and Methods

### 2.1. Animals and Phenotypes

Ethical approval was obtained from the University of Liverpool Research Ethics Committee for this study. The scientific procedures regulated by the Animals Act were performed under Home Office Project License PPL 70/8330, in compliance with relevant guidelines and regulations. The study adhered to the ARRIVE (Animal Research: Reporting of In Vivo Experiments) guidelines. The study included 554 Holstein–Friesian cows from three farms in North-West England and North Wales between October 2016 and June 2017. The trait under investigation was ketosis, measured using BHB detectors (FreeStyle Optium Neo H, Abbott Laboratories, United Kingdom) for 253 cows 3–4 weeks before the expected calving.

### 2.2. DNA Sampling, Extraction, and Genotyping

The methods for DNA sampling, extraction, genotyping, and quality control are described in Sánchez-Molano et al. [24]; briefly, a total of 554 cows were genotyped, with 266 animals using the Affymetrix Axiom Bovine 54K array and the remainder with the Illumina BovineSNP50 bead chip. Genotypes from the Affymetrix array were converted to the Illumina format by selecting 50,893 common single nucleotide polymorphisms (SNPs) based on consistent strand assignments and allelic calls. In the quality control steps, SNPs with a genotype call rate below 95% were removed to ensure high genotyping accuracy. Further filtering excluded markers with a minor allele frequency (MAF) less than 0.01 and those significantly deviating from the Hardy–Weinberg equilibrium, using a Bonferroni-corrected threshold of 1.48e − 6. At the sample level, individuals with a call rate below 95% were also excluded. The final dataset consisted of 253 animals and 31,204 SNPs, with positions based on the UMD 3.1 assembly.

Estimates of the variance components for the trait were obtained by using a linear mixed model in GCTA [25]. The model decomposes the phenotypic variance (*V*_p_) into genetic variance (*V*(G)) and environmental variance (*V*(e)). The following model was used:

 $$\begin{array}{c} y_{i}=\mu +G_{i}\beta +u_{i}+\varepsilon_{i}, \end{array}$$

where *y*_*i*_ represents the phenotype for individual *i*, *μ* is the overall mean, *G*_*i*_ denotes the genetic effect for individual *i*, *β* is the vector of fixed effects, *u*_*i*_ is the random effect due to genetics (assumed to be normally distributed with variance *V*(G)), and *ϵ*_*i*_ is the residual error (assumed to be normally distributed with variance *V*(e)).

To explore the genetic population structure of cows with recorded BHB levels, a multidimensional scaling (MDS) analysis was performed using the genomic relationship matrix (GRM). A dissimilarity matrix was computed by subtracting the GRM values from 1, which was then used to perform classical MDS using the cmdscale() function in R, extracting the first two dimensions. Phenotypic metadata, including farm of origin (farm), parity number, and body condition score (BCS), were matched to each individual and used to visually annotate the MDS plot. Farm was represented by color, parity by point shape, and BCS by point size.

Genome-wide association analyses were conducted using the genome-wide efficient mixed model association (GEMMA) [26] software as described in Sánchez-Molano et al. [24]. The trait assessed in the study was BHB measurements from freshly collected blood of animals. The fixed effects of farm, parity, and BCSs were included in the mixed model to account for their impact on ketosis. A Bonferroni-corrected significance threshold of 1.60e − 6 and a suggestive threshold of 3.20e − 5 were used in the Manhattan plot.

## 3. Results

**Figure 1:**
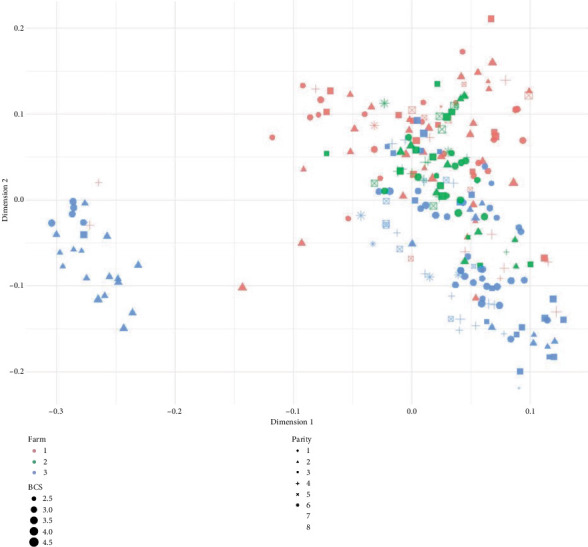
Multidimensional scaling (MDS) plot of 253 dairy cows based on genomic relationship matrix values. Each point represents an individual cow projected into two-dimensional space according to genetic similarity. Colors indicate farm of origin (red = Farm 1, green = Farm 2, and blue = Farm 3), shapes correspond to parity number, and point sizes reflect body condition score (BCS). The figure illustrates both genetic clustering and the distribution of animals across farms and physiological parameters.

The study involved a total of 253 dairy cows with recorded BHB levels, representing a diverse sample from three different farms. Specifically, the sample comprised 87 cows from Farm 1, 55 cows from Farm 2, and 111 cows from Farm 3, ensuring a varied representation of environmental and genetic backgrounds across the farms. The MDS plot derived from the GRM revealed clear genetic structure among the 253 cows. A distinct separation was observed along the first principal dimension, primarily differentiating animals from Farm 1 (red) and Farm 3 (blue), with Farm 2 (green) animals broadly distributed and overlapping the others (Figure 1).

Parity and BCS distributions appeared more dispersed across the genetic space, with no strong clustering based on parity levels. BCS values were spread across all regions of the plot, indicating that variation in BCS does not align strongly with underlying genomic structure in this cohort. Overall, the MDS plot supports the inclusion of farm as a fixed effect in downstream analyses and suggests limited confounding of BCS or parity with genetic structure.

Estimates of the variance components are presented in Table 1. The heritability of the trait was calculated to be 0.047, with a standard error of 0.13.

**Figure 2:**
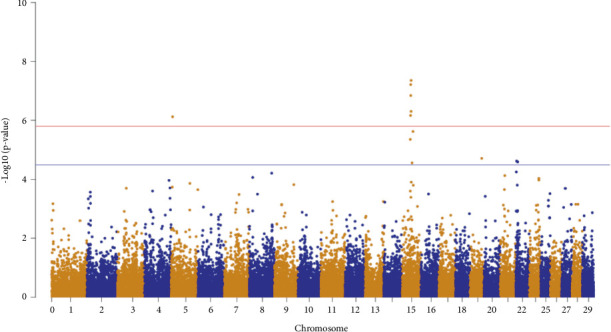
Genome-wide Manhattan plot. The red line indicates the Bonferroni-corrected significance threshold of 1.60e − 6. The blue line indicates the Bonferroni-corrected suggestive threshold of 3.20e − 5.

The GWAS conducted in this population identified six SNPs that surpassed the genome-wide significance threshold (Table 2). Five of these SNPs were located on Bos taurus autosome 15 (BTA15), spanning positions 39,063,079–41,597,038, and one SNP was identified on BTA5. Additionally, seven SNPs reached genome-wide suggestive significance, including three on BTA15, three on BTA22, and one on BTA19, as shown in Figure 2.

The genomic region defined by the significant SNPs on BTA15 contains several annotated genes, SPON1, FAR1 (fatty acyl-CoA reductase 1), PTH, BTBD10, ARNTL, RASSF10 (Ras association domain family member 10), TEAD1, PARVA, MICAL2, MICALCL, DKK3, and USP47, based on the UMD 3.1 assembly as referenced in the Ensembl genome browser.

## 4. Discussion

In the present study, genome-wide association analyses were used to assess the effect of genetics on BHB levels in cows before calving. The heritability estimate is approximately 4.7%, with a high standard error indicating considerable uncertainty. In comparison, other studies that have also used BHB measurements as the trait of interest have reported heritability estimates between 0.14 and 0.28 [27–30]. This low heritability suggests that genetic factors have a limited impact on the trait compared to environmental factors. Furthermore, the high *p* value means there is insufficient evidence to conclude that the genetic variance significantly contributes to the phenotypic variance. In summary, while the analysis indicates some genetic variance, the result suggests that genetic factors have a relatively small and nonsignificant impact on the phenotype. The high standard errors and *p* value highlight the need for cautious interpretation and possibly larger sample sizes for more reliable estimates.

The genomic region on BTA15 identified in this study aligns closely with a QTL previously characterized by Klein et al. [31] and harbors a group of genes implicated in lipid metabolism and obesity-related traits. Among these, *SPON1* encodes an extracellular matrix protein previously associated with body mass index [32] and obesity in murine models [33, 34]. FAR1 is critical for glycerophospholipid and ether lipid synthesis [35] and regulation of ether lipid synthesis [36]. The *PTH* gene, encoding parathyroid hormone, shows elevated expression in obese individuals, linking it to systemic lipid dysregulation [37–39]. Furthermore, *BTBD10* has been associated with body fat percentage in indigenous Alaskan populations [40], highlighting its potential role in adiposity. The ARNTL1 protein was shown to have effects on lipid metabolism and milk yield in Holstein cows [41, 42] and has also been implicated in obesity when its expression is disrupted in mice [43]. RASSF10 has not been reported to be associated with ketosis or related traits. However, members of the same protein family, specifically RASSF5 [17] and RASFF6 [2, 17], have been found to be linked with dairy cattle ketosis. Similarly, proteins from the ubiquitin-specific peptidase family, including USP2, USP4, USP19, and USP53, have shown associations with dairy cattle ketosis, suggesting a potential association for USP47 [17]. *TEAD1*, overexpressed in patients with metabolic dysfunction–associated steatotic liver disease, influences cellular lipid content, with its knockdown leading to significant reductions [44]. Additionally, MICAL2, encoding a monooxygenase, is upregulated in response to a high-fat diet, and its overexpression suppresses adipogenesis [45]. Finally, hepatic expression of *DKK3* is linked to obesity and fatty liver disease [46], and genetic polymorphisms in this gene are associated with increased back fat thickness in pigs [47]. The involvement of these genes in lipid metabolism suggests a potential mechanistic link to ketosis in dairy cattle, where lipid dysregulation plays a critical role in disease progression.

In conclusion, this study provides preliminary insights into the complex role of genetic factors in the development of ketosis in dairy cattle. Our findings suggest that specific loci, particularly on BTA15, may influence susceptibility to ketosis through pathways related to lipid metabolism. However, the relatively low heritability estimate indicates that environmental and management factors likely contribute substantially to phenotypic variation. Moreover, while the use of a single trained assessor may have reduced variability during data collection, it also introduces the possibility of operator-specific bias, which may limit the broader reproducibility and robustness of the findings. These results, though exploratory, emphasize the value of integrating genomic information with improved herd management strategies. Such a combined approach could support the development of more sustainable and resilient dairy production systems that promote both animal health and economic efficiency.

## Figures and Tables

**Table 1:** Estimates of heritability and variance components (*V*(G): Genetic variance, *V*(e): Environmental variance, *V*_p_: Phenotypic variance, *V*(G)/*V*_p_: Heritability (*h*^2^), SE: Standard error).

**Source**	**Variance**	**SE**
*V*(G)	0.008159	0.023123
*V*(e)	0.166332	0.026821
*V*_p_	0.17449	0.015563
*V*(G)/*V*_p_	0.046757	0.132249
*p* value	3.58e − 01	
*n*	253	

**Table 2:** Genome-wide significant SNPs. (The base pair positions are based on the UMD 3.1 assembly. The beta coefficient [representing the effect of minor allele substitution], its standard error, and the *p* value [*p*] for the beta coefficient are also provided).

**Chromosome (BTA)**	**rs**	**Position (bp)**	**Beta coefficient**	**p** ** value**
15	ARS-BFGL-NGS-4138	41,597,038	0.430 ± 0.076	4.40e − 08
15	Hapmap57927-rs29025966	39,724,096	0.443 ± 0.079	6.09e − 08
15	ARS-BFGL-NGS-69700	40,006,891	0.443 ± 0.082	1.44e − 07
15	Hapmap52590-rs29020878	41,383,202	0.347 ± 0.067	4.97e − 07
15	ARS-BFGL-NGS-11846	39,063,079	0.368 ± 0.072	6.85e − 07
5	ARS-BFGL-NGS-36420	6,786,633	0.310 ± 0.061	7.59e − 07

## Data Availability

The data that support the findings of this study are available from the corresponding author upon reasonable request.
